# Impact of different strains of *Bacillus* spp. on the bulb production of *Tulipa sintenisii* Baker

**DOI:** 10.3389/fpls.2024.1456919

**Published:** 2025-02-06

**Authors:** Ahmet Yenikalaycı

**Affiliations:** Plant Production and Technologies Department, Faculty of Applied Sciences, Mus Alparslan University, Mus, Türkiye

**Keywords:** *Bacillus* spp., Muş tulip, rhizobacteria, tulip bulb, *Tulipa sintenisii*

## Abstract

As an ornamental plant, *Tulipa sintenisii* (Muş tulip) has great potential for potting and cut- flowers in floriculture. However, its low number of bulb production per plant is a major constraint to it becoming one of the common cultivated tulip species. This study was conducted to determine the impacts of 10 *Bacillus* species on bulb number increase as well as other plant parameters of *T. sintenisii* in the Mus province of Turkey in the 2020/2021 growing season. Selected, equally sized *T. sintenisii* bulbs were soaked with *Bacillus* spp. solution (3.4 × 107 CFU/cm^3^) for 2 s, and the inoculated bulbs were planted in the experimental field in autumn. The experiment was organized in a completely randomized block design with six replications. The investigated bulb parameters were taken at their physiological maturity. The tulip bulbs treated with *Bacillus* spp. had higher plant height (28.6 cm), bulb number/plant (2.25), total bulb weight (14.7 g), central bulb weight (13.1 g), central bulb length (40.9 mm), and central bulb diameter (26.8 mm) than the control treatment. The *Bacillus* strain EZF13 had the highest bulb number while EZF104 had the highest total bulb weight, central bulb weight, central bulb length, and central bulb diameter. These findings suggest that *Bacillus* treatment has great potential to increase bulb number per plant as well as other bulb parameters of native tulip species *T. sintenisii*. At the same time, an environmentally friendly production model was put forward without fertilizer application with bacteria application in tulips. At the same time, since the application of bacteria increases the usefulness of plant nutrients in the soil, it can be effective in reducing both the costs and the negative effects of fertilizers on the environment with less fertilizer use.

## 1 Introduction

Tulips are garden and cut-flower favorites from the *Liliaceae* family that belongs to the genus *Tulipa* having 150−160 species originating from central Asia (Coskuncelebi et al., 2008). Tulips are known as the third most important segment of cut flowers worldwide (Jhon and Neelofar, 2006; Marasek-Ciolakowska et al., 2012). The tulip bulb production throughout the world continues to increase over the years. Tulips are generally propagated vegetatively since propagation by seeds requires a long time and effort.

There are many tulip (*Tulipa* spp.) species growing in Turkey’s natural flora. One of these species is *Tulipa sintenisii* Baker (Muş tulip), an endemic species. It usually blooms in late April and early May, and has a flowering period of approximately 15–20 days. A single flower is formed from each germinated *T. sintenisii* bulb. The distribution areas of *T. sintenisii* are generally uncultivated fields and flat meadow areas (Yenikalayci et al., 2019). *T. sintenisii* has thick, red, and shiny petals with 35–40-cm plant height, which is suitable for growing as cut flowers, for potting, and as border plants.

Tulip bulbs are planted in autumn when the temperature drops and the flower stem begins to elongate with increasing air temperatures (approximately 14°C –20°C) at the beginning of spring. When the leaves dry completely, physiological maturation processes begin in the bulbs. During these development stages of the plant, while the main bulb begins to dry, the development of the baby bulbs reaches the highest level. In late spring, the upper part of the plant dries completely and after this period, the physiological maturation stages of tulip bulbs occur (Van Tuyl and Van Creij, 2007). During the physiological maturity process, leaf blades and male and female organs are formed in the internal structure of tulip bulbs. The completion of the physiological formation stage of the tulip, also known as the ‘G’ developmental stage, is one of the necessary processes for the flowering of tulip bulbs (De Hertogh and Le Nard, 1993). Although there have been many studies carried out to shorten the breeding process (Fortanier, 1973; Kuijpers and Langens-Gerrits, 1997; Ghaffoor et al., 2004), it takes approximately 5–6 years to propagate well-developed tulip bulbs by seeds (Zhang et al., 2023).

Beneficial soil microorganisms, a major part of the natural ecosystems, can stimulate plant growth and development, consequently, increasing the yield and quality of crops, and contributing a considerable amount of mineral solubility that can be easily adsorbed by the crop plant (Weller, 1988; Joshi et al., 2006; Balla et al., 2022). Due to the adverse impact of artificial fertilizers on human health and the environment, beneficial soil microorganism usage has been increasing globally in sustainable crop production systems (Cakmakci et al., 2007) since they promote plant growth and development, maintain or improve soil function and structure, enhance bioaccumulation and biogeochemical cycling of inorganic compounds, and control or inhibit plant pathogen growth and development (Doran and Zeiss, 2000; Ehrlich, 1990). In the rhizosphere of nutrient-deficient soils, nutrient mobilization and transformation highly depend on plant and microorganism interactions. At present, the use of beneficial soil microorganisms has become popular as a supplement to chemical fertilizers to have a satisfactory yield increase in sustainable crop production systems (Sturz et al., 2000). To enhance crop productivity, several symbiotic (*Rhizobium* sp.) and non-symbiotic bacteria have been used worldwide (Burd et al., 2000; Cocking, 2003). Among the huge number of microorganisms, *Acinetobacter, Alcaligenes, Azospirillum, Azotobacter, Bacillus, Burkholderia, Derxia, Enterobacter, Gluconacetobacter, Herbaspirillum, Klebsiella, Lysobacter, Paenibacillus, Pseudomonas*, and *Rahnella* have great potential as growth promoters or biofertilizers (Rodriguez et al., 2006).

The beneficial effects of *Bacillus* spp. inoculations on plant growth and development have been attributed to the production of plant growth regulators, enzymes, and natural antibiotics as well as biological control properties and antagonism effects against phytopathogenicity (Subiramani et al., 2020; Chandran et al., 2021; Etesami et al., 2021; Mahapatra et al., 2022; Ortiz and Sansinenea, 2022; Shen et al., 2023; Khoso et al., 2024). In addition to these, the growth and development of the root system of crop plants are improved by *Bacillus* spp., consequently, they enhance water and nutrient absorptions (Hungria et al., 2013; Ji et al., 2014; Galindo et al., 2018). Therefore, bulb number and bulb weight could be increased with the application of *Bacillus* spp.

Unlike other tulip species, the ability of *T. sintenisii* to produce daughter bulbs is very low. Bulb size and weight are characteristics that directly affect tulip flower quality and size. This situation is a factor that limits the cultivation and commercial production of *T. sintenisii*. Beneficial soil microorganisms can improve the growth and development of *T. sintenisii* by supplying nutrients, producing growth hormones, improving soil structure, and inhibiting pathogens. Therefore, the aim of this study was to investigate the effects of different *Bacillus* spp. strains on bulb number, size, and weight of *T. sintenisii*.

## 2 Materials and methods

The tested *Bacillus* species were obtained from the Department of Agricultural Biotechnology, Erciyes University (**Table 1**). *Bacillus* species were grown in Tryptic Soy Broth (pH 7.0) (Merck, Germany) under aerobic conditions at 30°C with shaking at 250 rpm for 48 h. Each bacterial solution was prepared and the number of bacteria in the solution was adjusted to 1 × 10^9^ colony-forming unit CFU mL^-1^.

**Table 1:** The tested *Bacillus* species.

Bacteria	Nomenclature
EZF12 *B. simplex* EZF13 *B*. spp. EZF15 *B. nitrotireducens* EZF45 *B. cereus* EZF47 *B. subtilis* EZF73 *B.* spp. EZF84 *B. cereus* EZF96 *B. subtilis* EZF104 *B. cereus* EZF108 *B.* spp. Control	SY29.1 (MH853359.1) (in firmicutes) strain KH16.2 (MH847787.1) PSY1 (MW193119.1) PSY6.2.A (OK384682.1) KH28.1(MH846613.1) KH6.4 Group sp strain PSY6.2A (OK384684.1) KH(18.2 (MH853353.1) Group strain SY10.1A (OK384686.1) KH6.3A

The *T. sintenisii* bulbs were obtained from Muş Alparslan University, Tulip Research Center. Several *T. sintenisii* plant selection programs were completed at Muş Alparslan University, Tulip Research Center. However, none of the selected clones of *T. sintenisii* has not been registered yet and there has not been any registered variety present in the market. Therefore, *T. sintenisii* bulbs used in the current study were a clone of selected *T. sintenisii*.

This experiment was conducted in the Experimental Field of Muş Alparslan University, (38°77′38”N latitude and 41°42′77”E longitude at an elevation of approximately 1,243 m above sea level). The soil of the experimental plots was a deep well-drained clay silt loam with a pH of 6.61, 2.21% organic matter, 22.1 kg ha^-1^ available phosphorus, and 780 kg ha^-1^ available potassium. Based on soil analysis, fertilizer was applied and incorporated into the soil prior to planting at a rate of 40, and 40 kg ha^-1^ N and P, respectively. Monthly maximum and minimum temperature, monthly average temperature (°C), monthly average relative humidity (%), and monthly total precipitation (mm) values of the experimental year are provided (**Table 2**). Total precipitation during the growing season was 385 mm and no irrigation was applied.

**Table 2:** Meteorological data in the Mus province, Turkey during the experiment.

Month	Maximum temperature (°C)	Minimum temperature (°C)	Average temperature (°C)	Average relative humidity (%)	Total precipitation (mm)
November	22.8	-3.3	6.5	69.6	38.2
December	8.6	-13.2	0.0	84.4	16.6
January	9.6	-25.8	-5.5	85.0	94.0
February	13.2	-7.9	-0.3	80.8	49.8
March	14.7	-7.0	3.9	69.8	166.4
April	27.0	2.2	13.6	48.8	7.8
May	32.2	5.9	18.7	39.9	11.6
June	36.4	9.6	23.4	26.7	0.6

The *T. sintenisii* bulbs were obtained from Muş Alparslan University, Tulip Research Center. The tulip bulbs were washed under tap water and dried on filter paper 1 day before bacterial treatment. The bulbs were soaked with bacteria solution (3.4 × 107 CFU/cm^3^) for 2 s, and then the bulbs were immediately planted at a rate of 4 bulbs/m per row and 15-cm deep on 3 November 2020 and on 7 November 2021. The design of the experiment was a completely randomized block with four replications. The tulip bulbs were planted in a four-row 6-m long plots. The inter-row and intra-row spacing were 0.35 m and 0.25 m, respectively. The height of each plant in the two middle rows was measured at the flowering stage on 28 April 2020. At physiological maturity, all plants were dug up in the two middle rows on 10 June 2021. After digging, the bulbs on each plant were dissected into daughter bulb and central bulb.

The plant height (cm), number of bulbs per plant, total bulb weight (g), central bulb weight (g), and central bulb length and diameter (cm) were measured.

Field data were subjected to one-way analysis of variance using the GLM procedure of the SAS statistical package version 9.0. Means were compared using Fisher’s protected least significance difference (LSD) at type-I error of 0.05.

## 3 Results

Data from 2020 and 2021 trials were combined and analyzed together because there were no significant differences between the two cropping seasons for all of the investigated plant parameters.

**Figure 1:**
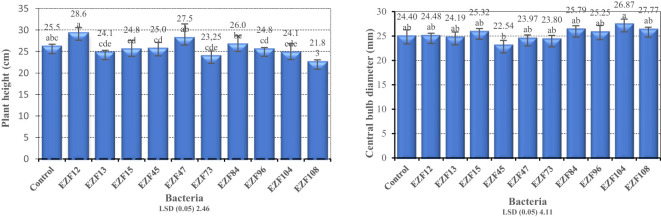
The plant height and central bulb diameter of Muş tulip treated with different *Bacillus* species. Different letters indicate significant differences at p < 0.05.

Among the mature bulbs treated with different *Bacillus* species, significant differences (P > 0.05) were observed for plant height and central bulb diameter (**Figure 1**).

The highest plant height was obtained from EZF12*-*treated plants with 28.6 cm followed by EZF47 and the lowest was obtained from EZF108-treated plants with 21.8 cm (**Figure 1**). Seven of the plants treated with *Bacillus* spp. had the lowest height than the control treatment.

The effects of *Bacillus* on the central bulb diameter of Muş tulip were significant (**Figure 1**). Central bulb diameter varied between 22.5 and 26.8 mm. Plants treated with EZF45 B. spp. had the lowest central bulb diameter while plants treated with EZF104 B. spp. had the highest central bulb diameter.

**Figure 2:**
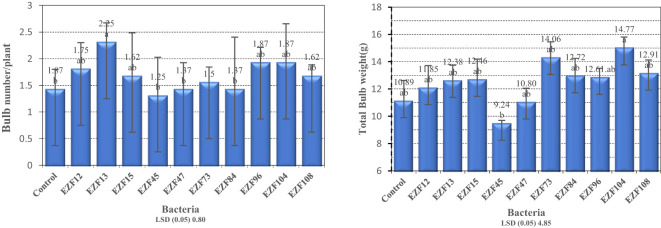
Bulb number/plant and bulb weight of Muş tulip treated with different *Bacillus* species. Different letters indicate significant differences at p < 0.05

Differences in bulb number per plant were significant among *Bacillus* spp. treatments (P < 0.05; **Figure 2**). Bulb number per plant varied between 1.25 and 2.25, the maximum bulb number per plant was obtained from EZF13 and the minimum was obtained from EZF45. Except for EZF45, all treated bacteria species had significantly higher bulb number per plant compared with the control treatment. Under natural growth conditions, *T. sintenisii* generally produces one bulb per year. Seven of the *Bacillus* species increased the bulb number per plant. When bulb production was the main target, EZF13 treatment could be the best application to increase bulb number per plant.

A similar trend was observed for total bulb weight. Analysis of variance showed a significant total bulb weight difference among *Bacillus* spp. treatments (P < 0.05; **Figure 2**). Total bulb weight varied between 9.2 and 14.7 g. The heaviest total bulb weight was obtained in EZF104-treated bulbs and the lightest bulb was recorded in EZF45-treated bulbs. Eight of the *Bacillus* species-treated bulbs had greater total bulb weight than the control treatment.

**Figure 3:**
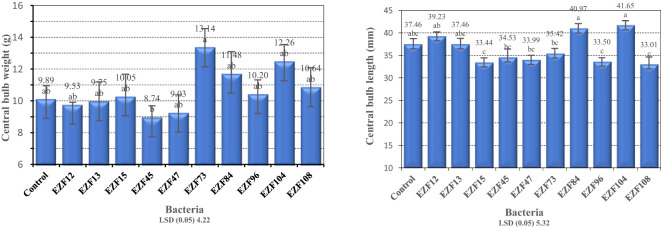
The central bulb weight and central bulb length of Muş tulip treated with different *Bacillus* species. Different letters indicate significant differences at p < 0.05

When central bulb weight was in consideration, EZF73 had the highest central bulb weight with 13.14 g followed by EZF104 and EZF84. The lowest central bulb weight was noted in treatment EZF45 with 8.74 g followed by EZF47 (**Figure 3**).

The tested *Bacillus* species differed significantly (*P* ≤ 0.05) from each other in their response in terms of central bulb lengths (**Figure 3**). The central bulb length varied between 41.6 and 33.0 mm. The highest and the lowest central bulb lengths were obtained from EZF104 and EZF108, respectively. The results showed that *Bacillus* treatment increased the bulb diameter. Three of the bacterial treatments (EZF104, EZF84, and EZF12) had higher bulb lengths than the control treatment.

The results of the present study showed that *Bacillus* spp. treatment had a positive impact on central bulb diameter. Compared with the control, six of the bacterial treatments increased bulb diameter (**Figure 3**). However, only the inoculation of EZF104 significantly increased bulb diameter. The treatment EZF45 had significantly lower bulb diameter than the control treatment.

## 4 Discussion

The improvement in the measured bulb parameters of *T. sintenisii* cannot be attributable to a single factor alone. The inoculation of the strains of *B.* spp. BEZF13 and *B. cereus* EZF104 showed the best effect on the evaluated *T. sintenisii* bulbs. It is well known that plant growth-promoting rhizobacteria are capable of promoting plant growth and development by synthesizing phytohormones, and enhancing plant nutrient acquisition and utilization (Corrales et al., 2014; Chandran et al., 2021; Etesami et al., 2021; Mahapatra et al., 2022; Ortiz and Sansinenea, 2022; Khoso et al., 2024). They are widely used as growth enhancers for many crop plants (Blake et al., 2021; Dobrzyński et al., 2022; Youssfi et al., 2024; Vincze et al., 2024).

The ability of *Bacillus* spp. strains to colonize the bulb or root system depends on plant species (Yanti et al., 2017). Inoculated *Bacillus* spp. strains must be able to establish and interact with the root system (Blake et al., 2021; Rajer et al., 2022; Shen et al., 2023). Compared to control, the inclusion and colonization of *Bacillus* spp. strains EZF12, EZF47, and EZF84 on bulbs increased plant height by 12.2%, 7.8%, and 1.9%, respectively. The central bulb weight, length, and diameter are important bulb parameters to assess the potential of tulip bulb blooming in the next spring. In the current study, central bulb weight significantly increased with *Bacillus* spp. strain treatments. The size of bulbs and bulblets depends on tulip species and tulip cultivars (Kleynhans, 2006). The ideal bulb size for most of the tulip species is approximately 12 cm. However, for *T. sintenisii*, bulb size is approximately 4 cm. For all tulip species, larger bulbs are highly preferred since smaller bulbs have low crop quality with smaller flowers and shorter plant heights. In the current study, the bulb size of plants treated with EZF12, EZF84, and EZF104 *Bacillus* spp. strains were greater than the control plants. Central bulb weight was one of the most important bulb parameters significantly influenced by inoculation of the *Bacillus* spp. The size of tulip bulbs in order to flower varies by species; the minimum size is generally from 6 to 8 g. Carl et al. (2015) stated that this range corresponds to approximately 6 to 9 cm in circumference. Since tulip bulbs must reach a critical weight in order to have a flower bud (Sestras et al., 2007). In the present study, central bulb weight was increased 45.5% by inoculation of the *Bacillus* spp. strain EZF73. Total bulb weight was another parameter positively influenced by the *Bacillus* spp. Total bulb weight was increased 35.6% by inoculation of the *Bacillus* spp. strain EZF104. These results can be attributed to the success of these *Bacillus* spp. strains (EZF73 and EZF104) in associating with tulip roots. After planting tulip bulbs, bacteria inoculation or soil fertility will not affect blooming of the bulbs in the next spring, but it will influence the growth and development of new bulbs. Therefore, inoculation of tulip bulbs with proper strains of *Bacillus* spp. can increase the size and weight of tulip bulbs.

## 5 Conclusion

Tulip bulbs treated with *Bacillus* spp. showed improvement in plant height, total bulb number, and central bulb weight, length, and diameter. *Bacillus* spp. strain EZF13 has great potential to increase bulb number per plant. Tulip bulb treatment with this strain could be recommended to increase the bulb number of *T. sintenisii* under field conditions in commercial tulip bulb production. Determining the appropriate bacterial strains for each crop may be beneficial in terms of increasing soil fertility, reducing fertilizer rate, and decreasing the negative impact of fertilizers on the environment.

## Data Availability

The raw data supporting the conclusions of this article will be made available by the authors, without undue reservation.
